# An examination of online physical education participation and its effects on health promotion

**DOI:** 10.3389/fpubh.2025.1612710

**Published:** 2025-07-09

**Authors:** Yu-Tai Wu, Yu-Feng Wu, Jian-Hong Ye, Weiguaju Nong, Jhen-Ni Ye

**Affiliations:** ^1^Office of Physical Education, Soochow University, Taipei, Taiwan; ^2^Office of Physical Education, Ming Chi University of Technology, Taipei, Taiwan; ^3^Faculty of Education, Beijing Normal University, Beijing, China; ^4^School of Education, Guangxi University of Foreign Languages, Nanning, China; ^5^Graduate Institute of Technological and Vocational Education, National Taipei University of Technology, Taipei, Taiwan

**Keywords:** course engagement, health promotion, online course, physical education, physiological and psychological, educational contingencies, positive psychology

## Abstract

**Introduction:**

The COVID-19 epidemic significantly impacted global education patterns. To protect the health of students, many countries and regions implemented online curricula during the peak of the epidemic, allowing teaching to continue despite the suspension of classes. However, whether all subjects, such as physical education (PE), can be effectively learned online is worth exploring.

**Methods:**

To achieve the objective of this study, junior high school students in China were invited to fill out an online questionnaire via a snowball sampling method. The retrieved data were validated for reliability and validity, and the three types of online course engagement (behavioral, emotional, and cognitive) were validated through a structural equation model to explore the effects on physiological and psychological health promotion.

**Results:**

The results showed that not all types of online course engagement positively impacted physiological and psychological health promotion. Specifically, behavioral, cognitive, and emotional engagements were shown to positively influence students’ perceived physiological health. While emotional and cognitive engagements were also shown to positively influence perceived psychological health, behavioral engagement did not significantly impact psychological health promotion. In addition, the analysis of the quality of online PE courses identified five main influencing factors: (1) lack of learning based on real experiences, (2) insufficient peer interaction, (3) environmental interference, (4) poor internet connection, and (5) poor learning uptake.

**Discussion:**

The findings showed how students’ physical and mental health is promoted through behavioral, emotional, and cognitive engagement in the context of online physical education courses. At the same time, the major confounding factors identified will help physical education teachers to remove these influences and make them better at delivering online physical education programs.

## 1 Introduction

A pandemic triggered by the SARS-CoV-2 virus overwhelmed the world in beginning of 2020. The crisis of COVID-19 has significantly impacted both the physical and mental well-being of citizens globally. Students faced additional challenges due to the sudden and unexpected shift in the delivery of their education, requiring them to adapt to the “new normal” (1). Health concerns became a focus, taking precedence over all other worldly issues and enabling individuals to be proactive about their health. Knowledge on the factors that affect an individual’s health is central to health promotion, which was critical during this crisis (2).

Health can be identified as optimal spiritual, social, emotional, physical, and intellectual well-being, and health promotion is the art or science of facilitating others make lifestyle alterations to attain optimal health (3). Health promotion is an active process that empowers individuals to take greater control of their health (4). Historically, physical activity has been associated with a healthy mind, and exercise has been an essential component of education (5). Therefore, an appropriate course designed for physical activity should effectively promote students’ physiological and psychological health.

Physical education programs have traditionally been developed to promote students’ health worldwide. However, due to the COVID-19 pandemic, the sudden shift to a home learning model on 24 March 2020 forced all schools to transition to online delivery of course material (6). This change meant that nearly all students had to study at home, despite it not always being the best learning environment. It has been found that digital technologies can be used to extend coverage and provide access around the world, without actual face to face interaction (7). The barriers created by online home learning significantly reduced the quality of education and affected students’ motivation and engagement (8). The lack of adequate network equipment and teachers’ ill-preparedness for the switch further widened the digital divide. Therefore, understanding the factors that influence online learning engagement is essential for addressing these issues and providing equal opportunities for all students (6).

The need to understand online learning engagement stems from its increasing recognition as a key indicator of success in the classroom (9, 10). Student engagement involves their active participation in learning activities (11). Engagement can also be categorized into three types: behavioral, emotional, and cognitive, each describing distinct characteristics of how students act, feel, and think (12). Behavioral engagement (BE) includes participating in discussions during online courses, asking questions, and paying attention to the learning material. Emotional engagement (EE) refers to learners’ positive emotions toward the instructor, peer learners, and the online course itself. Cognitive engagement (CE) involves the cognitive effort that learners invest in acquiring complicated knowledge or abilities during their online course learning (13).

Effective learning expects students to enthusiastically engage in learning behaviors (14). When students are deeply engaged, they deposited more effort into meeting learning requirements by acquiring the knowledge or skills required to achieve their learning goals (15). However, few studies in existing literature have examined learning engagement during a pandemic (11). Therefore, to expand the understanding of online learning engagement and its benefits during the pandemic, this study examined how three types of learning engagement affect learners’ course learning outcomes (health promotion) in online physical education courses through the COVID-19 pandemic.

In the following chapters, the theoretical foundations are introduced, followed by the formulation of the research hypotheses and model. Then data collection is carried out through online questionnaire survey, followed by analysis of measurement model, the test for the reliability and the validity analysis, and fitness of model analysis Furthermore, hypothesized model validation is carried out and discussion of the results of the study with past literature, culminating in the conclusions and recommendations of the study, as well as the limitations of this study and suggestions for future research.

## 2 Theoretical foundations, research hypotheses and models

### 2.1 Engagement theory

Engagement is an important variable in educational psychology for intensified focus and enhanced academic performance (16). Prior studies (17) highlight that such virtual communities not only enhance engagement but can also positively influence academic performance (AP) by encouraging shared learning and support. Educational technologies are recognized as providing learners with access to high-quality education, and when used appropriately, these technologies can enable learners to learn anytime, anywhere in a virtual environment, creating a meaningful learning experience for them (18). According to Sun et al. (19), each dimension’s mutual terms though distinct interact to create a solid structure in a multi-dimensional model. In this study, behavioral engagement (e.g., participation, effort, involvement in physical education) is deemed one of these interconnected dimensions. While behavioral engagement focuses on observable actions, it is closely tied to other facets such as emotional engagement and cognitive engagement. Together, these dimensions form a unified construct of engagement, reflecting the universal experience of participants in health-promoting physical education settings.

Behavioral engagement refers to an individual’s capability to finish the task of learning in an precise and accountable manner, emotional engagement demonstrates individuals’ sense of identification with and emotional response to the learning task throughout the learning process, and cognitive engagement is defined as the participants’ application of hypothetical cognitive strategies in the learning process (20). In the field of education, engagement theory is mainly concerned with the degree of students’ commitment to a particular learning/curriculum goal, which can be manifested in several ways such as affective, behavioral, and cognitive.

### 2.2 Research hypotheses

#### 2.2.1 The relationship between course engagement and physiological health promotion

Physical education serves as a primary tool for promoting health through sports. For many educators, health information is intricately linked with school sports lobbying activities (21, 22). Engagement in physical activity is typically endorsed due to its beneficial effects on physical fitness (23) and health (24). In addition, physical education has long been recognized as the primary avenue for health promotion through sports, with health information being closely intertwined with school sports lobbying activities in the minds of many educators (21). The original structure of the physical education system has been shown to improve the quality of students’ physical education, fostering well-developed health and fitness abilities necessary to sustain high levels of performance in professional activities (22, 25). However, in the digital learning environment, students often interact more with online learning systems and are distanced from their instructors and peers, potentially impacting their engagement and subsequent outcomes (8).

When learners are engaged, they are inclined to devote more time, energy, and cognitive resources to the task at hand (10). Engagement is recognized as an important factor in student learning achievement and success. Researchers have identified engagement as a construct that is multidimensional which encompasses various factors of active student involvement and commitment to the learning process (26). Behavioral, emotional, and cognitive engagement collectively defines a person’s engagement in an activity. The absence of any of these dimensions may indicate a lower degree of involvement or investment in the activity. For example, individuals who participate behaviorally in an activity but lack emotional or cognitive investment are considered to have lower engagement (12, 27).

From the perspective of active learning, the engagement can be seen as a catalyst for the process of learning, where the achieved learning outcomes support individuals’ commitment to subsequent steps (28). Positive emotions often accompany high levels of engagement, and individuals with heightened engagement tend to exhibit better psychological and physiological health than those with lower engagement levels (29, 30). When students are confronted with numerous online platforms and courses, their level of engagement in online physical education seems to depend on their behavioral, emotional, and cognitive engagement (31). This study utilized three types of course engagement to explore the relationship between participants’ physiological health promotion brought about by online physical education courses. The following hypotheses are shown below:

*H1*: Behavioral engagement positively impacts physiological health promotion.

*H2*: Emotional engagement positively impacts physiological health promotion.

*H3*: Cognitive engagement positively impacts physiological health promotion.

#### 2.2.2 The relationship between course engagement and psychological health promotion

A previous study has provided evidence that regular exercise over a period of as little as 2 weeks can alleviate symptoms of stress (32). According to the theory of behavioral confirmation, engagement can be experimentally manipulated and affect an individual’s psychological traits or state, as well as their abilities or actions (33). Some studies have demonstrated a strong relationship between emotional engagement and psychological health, including depressive symptoms and stress (34, 35). Additionally, several findings support the affirmative advantages of exercise on psychological health (36–38). Moreover, another study has validated that cognitive engagement is positively related to psychological well-being (39). Hence, it is evident that both emotional and cognitive engagement are important predictors of psychological health promotion.

Fredricks et al. (12) defined three dimensions comprising engagement: behavioral engagement (e.g., compliance, attendance, and involvement), cognitive engagement (e.g., investment in an activity and appreciation of its challenges), and emotional engagement (e.g., positive responses such as enjoyment and a sense of belonging). Thus, in the context of online physical education courses, the digital learning process is of major concern. Students interact more with online learning systems, distanced from their instructors and peers, which may affect their engagement and subsequent outcomes (8). It is noteworthy that studies have confirmed that learners with positive emotions tend to exhibit better psychological and physiological health when they have higher levels of engagement when compared to those with lower levels of engagement (29, 30). It is important to understand how engagement affects learners’ psychological health (40, 41) and whether different types of engagement affect psychological health promotion (42, 43).

In this study, we hypothesize that students with lower behavioral, emotional, and cognitive engagement in online physical courses might be more likely to experience poor psychological health promotion. Conversely, students who are more behave orally, emotionally, and cognitively engaged in online physical courses are expected to experience better psychological health promotion. Therefore, the three types of course engagement were utilized in this study to investigate the association between participants’ perceptions of psychological well-being facilitated by online physical education courses, with the hypotheses found below:

*H4*: Behavioral engagement positively impacts psychological health promotion.
*H5*: Emotional engagement positively impacts psychological health promotion.
*H6*: Cognitive engagement positively impacts psychological health promotion.

### 2.3 Research model

**Figure 1:**
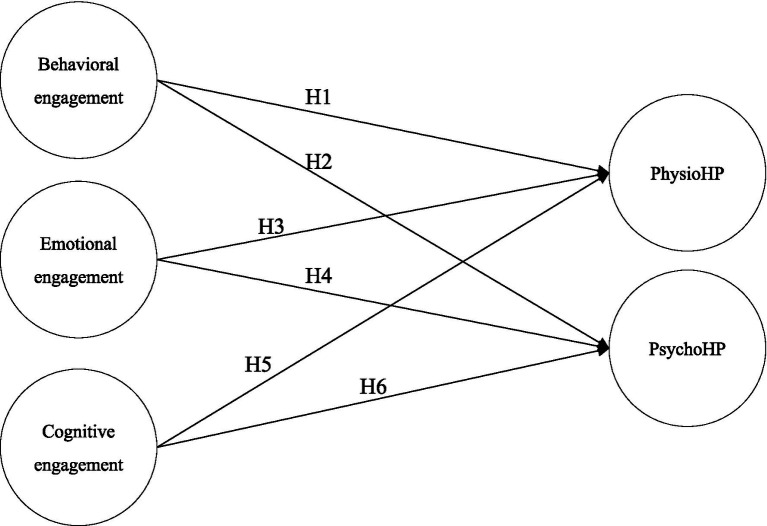
Research model.

Building upon the theoretical structure of learning engagement recommended by Fredricks et al. (12), this study examines the literature concerning the relationship between behavioral, emotional, and cognitive types of online course engagement and both physiological and psychological health promotion. Subsequently, the research proposes six research hypotheses formulated to construct a model, shown below (see Figure 1).

## 3 Research method

### 3.1 Research procedure

In this research, after obtaining research ethics approval from the institution of one of the authors. A snowball sampling method was employed to deliver an online questionnaire using the Wenjuanxing platform. Both QR code and questionnaire link were allocated on the WeChat and Weibo social media platforms, allowing Chinese secondary school students to access and fill out the survey. The initial page of the online questionnaire included a explanation of the study’s purpose, data collection and processing methods, participant anonymity protection, and informed consent details. Participants who completed the questionnaire were thought to have provided their informed consent. Upon anonymous finishing of the survey, they were encouraged to forward the questionnaire link to peers and friends. Responses were collected from a total of 436 questionnaires.

### 3.2 Participants

Fifty-eight of the 436 responses collected in the present study were invalid due to incomplete answers. Thus, the number of valid participants was 378 (86.7%). Among these, 180 (47.6%) were male and 198 (52.4%) were female. The participants’ distribution by academic year was as follows: 117 (31%) were in the first year of junior high school, 213 (56.3%) were in the second year, and 48 (12.7%) were in the third year. The mean age of the participants was 13.79 years, with a standard deviation of 0.60 years.

The distribution of the geographical locations of the participants was as follows: 94 (24.9%) were from Eastern China, 68 (18.0%) from Northern China, 64 (16.9%) from Central China, 4 (11.9%) from Southern China, 54 (14.3%) from Northeast China, 32 (8.5%) from Northwest China, and 21 (5.6%) from Southwest China. Regarding devices used for online learning, 14 (3.7%) used cell phones, 78 (20.6%) used tablets, 259 (68.5%) used computers, and 27 (7.1%) used smart TVs. Additionally, 102 (27%) participants preferred online physical education courses, while 276 (73%) disliked them.

### 3.3 Questionnaire development

A quantitative verification study was conducted to collect data using a questionnaire. The initial questionnaire was translated from English into Chinese. Three subject-matter experts in online physical education reviewed the translated questionnaire to confirm the accuracy of the translation and the correctness of the content, thereby establishing face validity. The a 5-point Likert scale, ranging from 5 (strongly agree) to 1 (strongly disagree) of the questionnaire survey was used. In addition, a group of seven Chinese second-grade students pretested the questionnaire. Based on their comments and feedback, the narrative of the questionnaire was revised to ensure all questions were easily understood by Chinese middle school students in the region. Moreover, a question regarding the external factors that affected the quality of learning in online physical education courses was included for students to select, aiming to investigate participants’ perceptions of the factors that affected the quality of their online physical education courses.

#### 3.3.1 Course engagement

Engagement in the learning process may be assessed as a form operating at the emotional, cognitive, and behavioral levels (12). Behavioral engagement involves attention and effort, while cognitive engagement encompasses regulation, monitoring, planning, and organization. Emotional engagement refers to interest and psychological behavior (44). The present study adapted the three types of course engagement scales from Luan et al. (45). An example of behavioral engagement could be a student stating, “I usually enter the online classroom on time when I am doing online physical education.” An example of emotional engagement could be a student stating, “I like to discuss sports-related issues with my classmates or teachers.” An example of cognitive engagement could be a student stating, “I remind myself to pay special attention to the areas where I tend to make mistakes in my sports posture.”

#### 3.3.2 Physiological health promotion

This study uses the guidelines from the General Administration of Sport (46) on fitness for all, the Health Promotion Administration, and the Ministry of Health and Welfare’s (47) physical activity guidelines for all to confirm the benefits of physical activity for physiological health, including strengthening and maintaining fitness. Eight questionnaire items were designed, including: “Participating in online physical education courses has helped me to become more physically fit” and “Participating in online physical education courses makes me more satisfied with my physical condition.”

#### 3.3.3 Psychological health promotion

This study used the guidelines from the General Administration of Sport (46) on fitness for all, the Health Promotion Administration, and the Ministry of Health and Welfare’s (47) physical activity guidelines for all to confirm the benefits of physical activity for psychological health, including enhancing positive emotions and relieving stress. Eight questionnaire items were designed, including: “I feel happier when I participate in online sports classes” and “Participating in online physical education courses makes me more confident.”

## 4 Results

After data collection, invalid data, such as incomplete responses or incorrectly filled-out personal background information, were first removed. Subsequently, the reliability and validity of the constructs and questionnaire content were examined through validation factor analysis and structural equation modeling.

### 4.1 Item analysis

The items was analyzed with first-order confirmatory factor. Studies have suggested that the value of *χ*^2^/df should be to a lesser extent than 5; RMSEA should be to a lesser extent than 0.10; GFI and AGFI ought to be greater than 0.80; and questions with factor loadings (FL) lesser than 0.50 ought to be removed from the original questionnaire (48, 49). Based on these criteria, items were removed from the questionnaire as follows: behavioral engagement was deleted from 6 to 5, emotional engagement from 6 to 5, cognitive engagement from 6 to 4, physiological health promotion from 8 to 6, and psychological health promotion from 8 to 6 (Table 1).

**Table 1:** First-order CFA.

Index	Threshold value	BE	EE	CE	PhysioHP	PsychoHP
*χ* ^2^	–	17.40	10.30	5.40	29	28.6
*df*	–	5	5	2	9	9
*χ*^2^/*df*	<5	3.48	2.06	2.70	3.22	3.18
RMSEA	<0.10	0.8	0.5	0.7	0.8	0.8
GFI	>0.80	0.98	0.99	0.99	0.98	0.98
AGFI	>0.80	0.95	0.97	0.97	0.95	0.94

### 4.2 Reliability and validity analysis

After the item analysis, the internal consistency and reliability of the scale was established using Cronbach’s *α* and composite reliability (CR), respectively. According to Hair et al. (48), Cronbach’s α and CR over 0.70 to be deemed acceptable. The Cronbach’s α values of this research were between 0.82 to 0.92 and the CR values were between 0.83 and 0.89 (Table 2), thereby meeting the suggested criteria.

**Table 2:** Reliability and validity analysis.

Construct	*M*	*SD*	α	*FL*	CR	AVE
BE	3.61	0.55	0.83	0.65–0.76	0.84	0.50
EE	3.50	0.64	0.85	0.66–0.77	0.85	0.53
CE	3.61	0.61	0.82	0.60–0.81	0.83	0.54
PhysioHP	3.76	0.67	0.90	0.69–0.74	0.87	0.52
PsychoHP	3.63	0.71	0.92	0.71–0.80	0.89	0.58

Convergent validity was determined by factor loading (FL) and average variance extracted (AVE). Hair et al. (48) indicated that the FL value must be greater than 0.50, with items less than this threshold being deleted. All the retained items in this study met these criteria. The FL values for behavioral engagement were between 0.65 to 0.76, emotional engagement was between 0.66 to 0.77, cognitive engagement was between 0.60 to 0.81, physiological health promotion was between 0.69 to 0.74, and psychological health promotion was between 0.71 to 0.80 (Table 2). Hair et al. (50) implied that the AVE value must not be lower than 0.50 to demonstrate convergent validity. The AVE values in this study was between 0.50 and 0.58 (Table 2).

Awang (51) specified that if the square root of the AVE value of all construct is superior than the Pearson correlation coefficient value of other constructs, the construct is considered to have discriminant validity. The outcome of the analysis showed that every constructs had discriminant validity (Table 3).

**Table 3:** Discriminant construct validity.

Construct	1	2	3	4	5
1. BE	(0.71)				
2. EE	0.56	(0.73)			
3. CE	0.63	0.61	(0.73)		
4. PhysioHP	0.66	0.60	0.60	(0.72)	
5. PsychoHP	0.51	0.64	0.63	0.61	(0.76)

The values along the diagonal are the square root of AVE, and the other values are for the related coefficients.

### 4.3 Model fit analysis

AMOS 20.0 was used in this study for structural equation modeling verification. Corresponding to the guidelines of Hair et al. (48) and Abedi et al. (52), the suggested values for each fitness metric are as follows: the *χ*^2^/df value should be to a lesser extent than 5, the RMSEA value must be to a lesser extent than 0.1, GFI, AGFI, NFI, NNFI, CFI, IFI and RFI must all be superior than 0.80, and PNFI and PGFI must be superior than 50. The fit index values for the overall suitability analysis in this study were: *χ*^2^ = 951.93, df = 293, *χ*^2^/df = 3.25, RMSEA = 0.08, GFI = 0.84, AGFI = 0.81, NFI = 0.84, NNFI 0.87, CFI = 0.89, IFI = 0.89, RFI = 0.83, PNFI = 0.76, and PGFI = 0.70.

### 4.4 Path analysis

**Figure 2:**
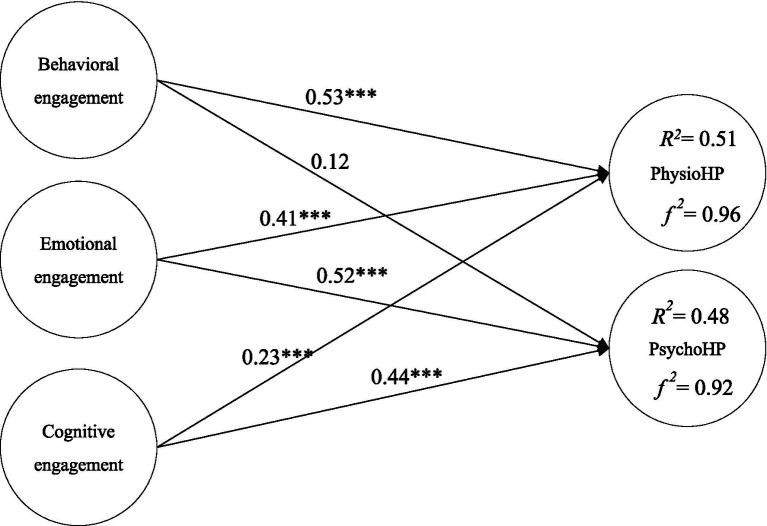
Validation of the research model. ***p < 0.001.

Model verification results showed that behavioral engagement had a positive effect on physiological health promotion (*β* = 0.53***) but not on psychological health promotion (*β* = 0.12). Emotional engagement had a positive impact on physiological health promotion (*β* = 0.41***) and psychological health promotion (*β* = 0.23***). In addition, cognitive engagement had a positive impact on physiological health promotion (*β* = 0.52***) and psychological health promotion (*β* = 0.44), (see Figure 2). The explanatory power of the three types of course engagement (behavioral, emotional, and cognitive) for physiological health promotion was 51%, with an *f*^2^ of 0.96. The explanatory power for psychological health promotion was 48% with an *f*^2^ of 0.92, (see Figure 2).

### 4.5 Analysis of external factors affecting the quality of learning in online physical education courses

In examining the number of external factors that affected the quality of learning in online physical education courses, each participant selected three external factors. The survey results indicated that the factors that affected the quality of online physical education courses were classified as follows: (1) lack of offline learning experiences, (2) insufficient peer interaction, (3) environment interference factors (e.g., noise), (4) bad internet connection, and (5) poor learning uptake (Table 4).

**Table 4:** External factors affecting the quality of online courses.

No.	Factors	Frequency (%)
1.	Lack of offline learning experiences	275 (24.3%)
2.	Insufficient peer interaction	268 (23.6%)
3.	Environment interference factors (e.g., noise)	147 (13%)
4.	Bad network connectivity	104 (9.2%)
5.	Poor learning uptake	89 (7.8%)
6.	Lack of physical teaching tools	78 (6.9%)
7.	Insufficient teacher-student interaction	66 (5.8%)
8.	Platform operation is not smooth	58 (5.1%)
9.	Other	32 (2.8%)
10.	Poor personal hardware	17 (1.5%)

### 4.6 Discussion

#### 4.6.1 The positive impact of the three types of course engagement on physiological health promotion

The global COVID-19 pandemic transformed the learning environment of students, thrusting both teachers and students into unexpected teaching and learning experiences. By March 2020, online delivery had become an increasingly effective and prevalent alternative to traditional classroom-based learning worldwide (53). The outcome of this study established that three types of course engagement positively influence physical health promotion. Students who perceive greater engagement (behavioral, emotional, and cognitive) in online physical education courses also perceive the program’s effectiveness in promoting physiological health more strongly.

Fredricks et al. (12) indicated that engagement can be explained in three ways. Behavioral engagement is evident through students’ adherence to academic standards, for example class presence, task completion, and independent participation in correlated activities. Emotional engagement worries students’ affective state in online courses, encompassing experiences such as anxiety, boredom, social belonging, and interest. Lastly, cognitive engagement denotes students’ focused involvement in deeper learning processes within the online learning environment.

The primary aim of this study was to determine how student’s engagement in online physical education courses influences physiological and psychological health promotion through behavioral, emotional, and cognitive engagement. As illustrated in Figure 2, all hypotheses were supported except for H2. Previous study showed belief in the psychological benefits of physical activity mediates the relationship between activity intensity and mental health outcomes. This implies that without a strong belief in the benefits, behavioral engagement alone may not lead to improved psychological health (54). It is now evident that students’ behavioral, emotional, and cognitive engagement directly predict both physiological and psychological health promotion. In addition, comparing the mean values of behavioral engagement (*M* = 3.61, *SD* = 0.55), emotional engagement (*M* = 3.50, *SD* = 0.64), and cognitive engagement (*M* = 3.61, *SD* = 0.61) outlines the significance of participants attribute to all three forms of engagements. Moreover, the mean scores for physiological health promotion (*M* = 3.76, *SD* = 0.67) and psychological health promotion (*M* = 3.63, *SD* = 0.71) were greater than 3, indicating a high level of engagement in both physiological and psychological health promotion among students.

Engagement in physical activity correlates with enhanced physical fitness (23) and better overall health (24). Physical education serves as a primary tool for promoting health through sports, with many educators associating health information with school sports lobbying activities (21, 22). Structured physical education programs contribute significantly to improving the quality of students’ physical education, fostering advanced health and fitness capabilities required in professional activities (25). Amidst the numerous online platforms and courses, students’ engagement resilience in online physical education courses depends largely on behavioral, emotional, and cognitive factors (31). Consistent with previous literature, this study presents evidence supporting the physiological health promotion effects of engagement.

#### 4.6.2 The effect of emotion and cognitive engagement versus behavioral engagement on psychological health promotion

Learning online can be particularly worrying for students, specifically individuals with lower levels of learning independence (55, 56). Many students have difficulty learning and interacting with others in an exclusively online environment for the initial time, leading to heightened worry at the onset of online courses (57–59). Therefore, it is vital to understand how students’ engagement in online physical education courses during the COVID-19 pandemic affects the promotion of their psychological health.

Previous research has highlighted the significance of engagement in schooling for students’ academic success and their development as competent members of society (60). Studies have also found that learners who are highly engaged in their courses receive better grades and experience better emotional regulation (34). In contrast, learners with lower levels of engagement are more probable to encounter academic disappointment and suffer from negative psychological consequences (61). In recent years, there has been increased attention on learning engagement, largely due to its association with learning outcomes (62, 63). Unlike traditional face-to-face learning settings, online interactions, including instructor-to-learner and peer-to-peer interactions, are facilitated by technology. Students who learn online are, therefore, more likely to feel cognitively or emotionally isolated compared to in-person learners (63). However, contrary to previous findings, this study reveals that students’ cognitive and emotional engagement positively correlates with psychological health promotion.

The study validates that emotional and cognitive engagement positively impacts psychological health promotion, consistent with previous studies (34, 35, 39). However, behavioral engagement does not significantly affect psychological health promotion. Thus, stronger perceptions of emotional and cognitive engagement in the online program correlate with stronger perceptions of the program’s effectiveness in promoting physical health. However, variations in perceptions of behavioral engagement do not significantly influence the perceived effectiveness of the online physical education program in promoting psychological health. Supporting previous research, this study provides evidence that engagement can be considered to have positive effects on psychological health promotion.

## 5 Conclusions and recommendations

### 5.1 Conclusion

This study constructed and verified a model using engagement theory to understand students’ physiological and psychological health promotion through behavioral, emotional, and cognitive engagement. Although the effect of the COVID-19 pandemic necessitated the adoption of online learning in mainland China in 2019, allowing for the continuation of courses without physical classes, existing studies on junior high school students have been limited in examining the benefits of online physical education programs. In addition, while former research have examined the positive effects of engagement on health promotion, the specific impact of different types of engagement on physiological and psychological health promotion has not been thoroughly examined. This study addresses this gap by investigating the strength of different types of engagement in the two types of health promotion.

This study examined the impact of course engagement in online physical education programs on health promotion among middle school students during the COVID-19 epidemic. The findings are as follows: (1) Three types of course engagement (behavioral, emotional, and cognitive) positively affect physiological health promotion. (2) Emotional and cognitive engagement positively influence psychological health promotion, while behavioral engagement does not have a significant effect. These results indicate that in order to effectively promote learners’ physiological and psychological well-being during an epidemic, teachers should focus on enhancing emotional and cognitive engagement through instructional design and strategies. However, it is also essential to monitor learners’ behavioral engagement, as it serves as the foundation for other types of engagement.

Moreover, current literature emphasizes that a lack of physical activity can have adverse health effects, potentially increasing risk factors associated with COVID-19. Therefore, maintaining a good level of physical activity is crucial (64). Using the COVID-19 pandemic as the research context, this study’s data analysis validates its findings, providing insight for teachers on how to improve the effectiveness of online physical education programs. These results also serve as a basis for advocating online physical education programs to promote health.

### 5.2 Recommendations

This study addresses the top five external factors that affected the quality of online physical education courses, including the lack of offline learning experience, insufficient peer interaction, environment interference (noise), bad internet connection and learning uptake. The interference refers to external factors in the learner’s surroundings that disrupt attention and hinder the students learning process which is unable for students to compete for the learner’s cognitive resources. Finally learning uptake refers to the learners absorb, process, and apply instructional content. It encompasses the learner’s engagement with the material and the degree to which they internalize and utilize the knowledge or skills given. The following recommendations for improvement are made:

To address the lack of offline learning experiences, teachers should avoid designing courses that require specific sporting facilities, such as ball games, outdoor sports, or swimming. Instead, courses should incorporate activities like aerobics, yoga, and basic physical training to facilitate home exercise. To mitigate insufficient peer interaction, teachers can include virtual interactive sports content, such as solitaire sports games, in their teaching design. Furthermore, digital material designers consider developing virtual reality or extended reality sports systems that allow multiple users to engage in individual or group sports without physical contact, thus enhancing peer interaction.

The issues presented by environmental interference (noise) and bad internet connection need to be collaboratively managed by teachers and students. Additionally, to create a quiet classroom environment, teachers should also remind students to mute their microphones before class. It is also necessary to check the network communication status prior to the class and minimize other high-network traffic usage during the lesson. Finally, to address poor learning uptake, El-Sayad et al. (8) highlighted the significance of online course teachers in not only imparting knowledge but also providing assistance and guidance to increase student engagement. Therefore, this study recommends that teachers do not assign excessively difficult exercises and offer flexible consultation methods and time. This approach ensures that students have ample time to seek help with physical education learning issues when they cannot see the teacher in person.

In addition, to enhance students’ emotional involvement and cognitive engagement in online sports lessons, teachers can utilize meta-universe technology to create immersive virtual sports games, which can stimulate students’ interest in learning and enhance their emotional involvement through highly simulated sports scenarios, so that students can obtain experiences similar to real sports in the virtual environment. In addition, conducting online sports competitions can create a competitive atmosphere, cultivate their teamwork and sportsmanship, and further enhance their emotional engagement. At the same time, the integration of rich online course resources provides students with diversified learning content and personalized learning paths, which helps to meet the learning needs of different students and promote their cognitive engagement. In terms of interactive tools, teachers can use cloud interactive tools to break the time and space limitations of traditional teaching and realize real-time and efficient interactive communication between teachers and students. Through cloud interactive tools, teachers can obtain students’ learning feedback in a timely manner, accurately grasp the students’ learning progress and difficulties, and then optimize teaching content and methods to achieve personalized teaching. For policy makers or teaching administrators, conferring to teaching requirements of online physical education courses, they should formulate corresponding professional development plans for teachers and organize training for online teaching toward physical education teachers, enabling teachers to master the methods the skillset for teaching online and improve their ability to teach on the Internet and the application of information technology. Detailed syllabuses and standards for online physical education courses can also be formulated, including teaching objectives, teaching methods, teaching planning, teaching evaluation, and so on, to ensure the quality of the courses.

### 5.3 Limitations and future study

This study is a quantitative verification study. Although the relationships between the hypothetical paths were verified, the factors influencing learning engagement were not included. Future studies should explore these factors further through qualitative interviews. This approach will enable teachers to provide more appropriate instructional designs and methods for online learning.

According to the influence of the pandemic during the data collection period, this study adopted the snowball sampling method as a contingency measure by collecting student questionnaires from different regions of China. Although this contingency measure has good practicality, it is prone to selection bias and the generalizability of the outcomes were restricted. Therefore, the sampling design can be based on the characteristics of the mother cohort to increase the inability of the results in subsequent studies. In addition, this study only focused on Chinese middle school students, and the results obtained may, to some extent, only be applicable to contexts such as China’s current educational environment, cultural background, and the specific technology access conditions in which the student population is embedded. Therefore, it is suggested that future studies may conduct cross-country (regional) comparative studies to gain a more comprehensive understanding of the similarities and differences in the relevant situations of secondary school students in different cultures, education systems and technological environments, as well as to broaden the scope of applicability of the research findings. This study offers a general overview of the effect of online physical education programs on health promotion without analyzing the effectiveness of these programs according to specific teaching themes, such as aerobics, ball games, and physical fitness. Future research should examine whether there are differences in the effectiveness of physiological and psychological health promotion across different types of teaching topics.

## Data Availability

The raw data supporting the conclusions of this article will be made available by the authors, without undue reservation.
